# Digital divide and health status among rural older adults in China: evidence from CHARLS

**DOI:** 10.1080/16549716.2026.2679945

**Published:** 2026-06-02

**Authors:** Weili Ren, Jianan Li, Yuge Tian, Deyan Liu, Zhiduo Chen, Shangjian Yang, Jie Ren

**Affiliations:** a School of Physical Education, Shandong University, Jinan, China; b Department of Thyroid Surgery, Taian Central Hospital, Taian, China; c School of Physical Education, Shandong Agricultural University, Taian, China

**Keywords:** Digital inequality, health disparities, rural ageing, cognitive function, social participation

## Abstract

**Background:**

Digital inequality may be associated with health disparities in rural ageing populations, yet its cognitive and social pathways and contextual conditions remain insufficiently understood.

**Objectives:**

To examine associations between a multidimensional digital divide and health status among rural older adults in China, evaluate indirect associations through cognitive function and social participation, and assess effect modification by household broadband access and physical activity.

**Methods:**

This was a cross-sectional secondary analysis of rural adults aged ≥60 years from the 2020 China Health and Retirement Longitudinal Study. Regression-based path models with bootstrapped confidence intervals were used to estimate total and indirect associations and interactions, with adjustment for sociodemographic covariates.

**Results:**

A larger digital divide was associated with poorer health status. In indirect analyses, cognitive function and social participation partly accounted for the observed association. Broadband access modified the digital divide-cognition association, with a weaker negative association among those with broadband access. Physical activity modified the social participation-health association, which was attenuated at higher activity levels.

**Conclusion:**

Digital inequality was associated with poorer health among rural older adults, with patterns consistent with cognitive and social pathways and variation by infrastructure and behaviour, suggesting potential targets for reducing digital health disparities.

## Paper Context

**Main findings:**

Digital inequality was associated with poorer health status among rural older adults, with indirect associations through cognitive function and social participation, and variation by household broadband access and physical activity.

**Added knowledge:**

This study extends previous work by examining cognitive and social pathways together and by considering infrastructure and behavioural context in rural ageing populations.

**Global health impact for policy and action:**

Improving reliable rural broadband access, practical support for using online health resources, and feasible community activities may help reduce digital health disparities among ageing populations.

## Background

Population ageing and digital transformation are reshaping health and social participation worldwide. In China, the number of adults aged ≥ 60 years is projected to rise substantially in the coming decades, intensifying pressures on health and long-term care systems [1]. At the same time, despite rapid growth in internet penetration, rural residents and older adults remain underrepresented online [2]. Older adults often face barriers in access and skills, and these constraints are typically more pronounced in rural settings where infrastructure, education, and social resources are limited [3]. Against this backdrop, understanding the association between the digital divide and health disparities among rural older adults, as well as its potential pathways, has become important for healthy ageing and equity-oriented policy.

The term digital divide initially referred to unequal access to digital technologies but has evolved to encompass gaps in skills, patterns of use, and the capacity to benefit from digital resources [4,5]. Digital inequality in later life cannot be reduced to a simple distinction between internet users and non-users. Among older adults, differences in the breadth of online activities and in practical digital skills remain important sources of inequality even after initial access has been achieved. Variation in the diversity and extent of online engagement is itself a meaningful dimension of digital inequality in older populations [6]. Likewise, digital inequality in middle-aged and older adults is shaped not only by access but also by differences in internet-related skills [7]. In line with this broader perspective, recent research in a Chinese sample has applied a multidimensional framework integrating access, usage, and application-related capabilities [8]. Guided by this line of research, the present study operationalized the digital divide through three observable dimensions available in CHARLS: digital access, diversity of online activities, and practical digital skills.

For older adults, a key downstream consequence is limited digital health literacy – the ability to locate, understand, appraise, and apply online health information and services – particularly among socioeconomically disadvantaged groups [9,10]. Rural older adults may therefore be less able to use digital channels for health information, appointments, payments, and social connection, potentially amplifying barriers to care and undermining both physical and mental health [9]. Against this background, we hypothesise that a larger digital divide is associated with poorer health status among rural older adults (H1).

### The mediating role of cognitive function

Cognitive function is a key resource for healthy ageing, and cognitive decline can progress from mild impairment to dementia, including Alzheimer’s disease [11]. Rural older adults often experience poorer cognitive health than their urban counterparts, partly due to structural constraints in education, resources, and stimulation opportunities [12]. Digital inequality may further widen this gap: limited access and low digital skills may reduce exposure to information, learning opportunities, and cognitively stimulating activities, especially those involving health information and services, and may be linked to poorer cognitive trajectories over time [13]. At the same time, poorer cognition can impede digital inclusion because older adults may struggle with interfaces, password management, and online instructions [14]. Taken together, we hypothesise an indirect association between the digital divide and health status through cognitive function among rural older adults (H2).

### The mediating role of social participation

Social participation is another core pathway to health in later life. Limited social relationships are associated with higher mortality risk and poorer self-rated health [15]. Digital technologies can help older adults maintain and expand social ties by enabling communication and access to social resources, thereby supporting engagement in offline and online interactions [16]. Evidence among rural older adults suggests that higher digital literacy is associated with stronger offline social interactions [17]. Accordingly, digital exclusion may be associated with fewer opportunities for social interaction and civic engagement, weaker social ties and support networks, and poorer health through reduced social participation. We therefore hypothesise an indirect association between the digital divide and health status through social participation among rural older adults (H3).

### The moderating role of broadband connection

Broadband connectivity may shape whether digital resources are associated with cognitive benefits. Digital exclusion can foster a low-stimulation lifestyle and reduce cognitively engaging activities, which may deplete ‘cognitive reserve’ and accelerate cognitive decline [18]. Stable household broadband can facilitate sustained online engagement, such as searching for information and communicating with others, and has been linked to greater health-information seeking than slower or less stable connections [19]. Recent longitudinal evidence from China further suggests that broadband access may support cognition indirectly by promoting internet use over time, and that increases in internet use are associated with more favorable cognitive trajectories among middle-aged and older adults [20].

Consequently, when digital inequality is more pronounced and barriers to access and use are greater, stable broadband may be more salient as a contextual condition that supports sustained online engagement and potentially broader social connection. Prior studies suggest that sustained digital engagement is associated with more active lifestyles and broader social networks, which are linked to better cognitive outcomes [21,22]. Prior research also shows that the breadth and depth of internet use are positively associated with cognitive function in middle-aged and older adults, suggesting that more stable access conditions may be especially relevant where cognitively stimulating digital engagement depends on sustained use [23]. Importantly, broadband connectivity is not synonymous with the digital divide: the digital divide, as operationalized in this study, is a broader multidimensional construct covering digital access, diversity of online activities, and practical digital skills, whereas broadband primarily captures the digital access/infrastructure dimension [24]. In rural settings, uneven broadband infrastructure is a key contributor to digital inequality [25], and mobile – only access may be less suitable for sustained information access and interaction due to limitations in stability, speed, and data costs [26]. We therefore hypothesise that broadband connectivity moderates the association between the digital divide and cognitive function (H4).

### The moderating role of physical activity

Physical activity may condition whether social participation translates into measurable health benefits. While the WHO recommends that adults aged ≥ 65 years accumulate 150–300 minutes/week of moderate-intensity activity or 75–150 minutes/week of vigorous-intensity activity [27], evidence increasingly highlights the value of light-intensity activity for physical and mental health in later life [28]. Evidence from studies of older adults further suggests that even light-intensity physical activity is positively associated with physical health and psychosocial well-being [29]. Beyond its direct association with health, physical activity may also reflect behavioural and functional capacity that shapes how older adults engage in, benefit from, and maintain social participation. Therefore, treating physical activity only as a confounder may underestimate its role in shaping health returns from social engagement [30,31]. In a prospective cohort of older adults, physical activity appeared to modify the effect of social participation on health-related outcomes, suggesting that the health implications of social participation may vary across activity levels [32]. We therefore hypothesise that physical activity moderates the association between social participation and health status among rural older adults (H5).

Although a growing literature links digital inequality to older adults’ health, the underlying mechanisms and boundary conditions remain incompletely understood. Many studies rely on single indicators (e.g. device ownership or internet/smartphone use) and focus on direct associations, with fewer testing both mediated pathways and contextual moderation – especially among rural older adults. Existing mediation evidence has largely emphasised social pathways such as health literacy, cultural capital [33], and social support [34], whereas cognitive and social pathways are rarely examined in parallel within the same model. To address these gaps, we treat the digital divide as the primary exposure and overall health status as the outcome among rural older adults in China. We posit two complementary pathways, cognitive and social, operationalised as parallel mediators: cognitive function and social participation. We further examine heterogeneity by introducing household broadband connectivity and physical activity as moderators. This framework may help identify potential intervention points for reducing digital health disparities through improvements in digital access and digital health literacy, alongside supportive behavioural conditions.

**Figure 1.**
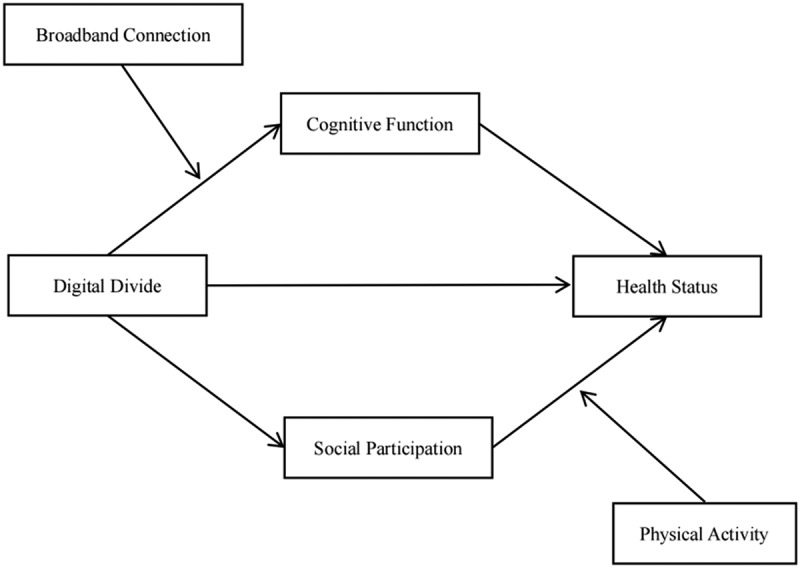
Hypothesized model.

Accordingly, the hypothesized research model is presented in Figure 1.

## Methods

### Study setting and sampling

Data were drawn from the 2020 wave of the China Health and Retirement Longitudinal Study (CHARLS), a nationally representative survey that collects microdata on Chinese households and individuals aged 45 years and older. The national baseline survey covered 150 counties and 450 communities (villages) across 28 provincial-level administrative units in China. This study was carried out in accordance with the principles of the Declaration of Helsinki.

### Research design and participants

Although CHARLS includes adults aged 45 years and older, the present study focused specifically on rural adults aged 60 years and above, because digital exclusion and health vulnerability are more pronounced in later life, particularly in rural settings. Accordingly, this study aimed to examine the association between the digital divide and health status among rural older adults, rather than across the full CHARLS – eligible sample.

**Figure 2.**
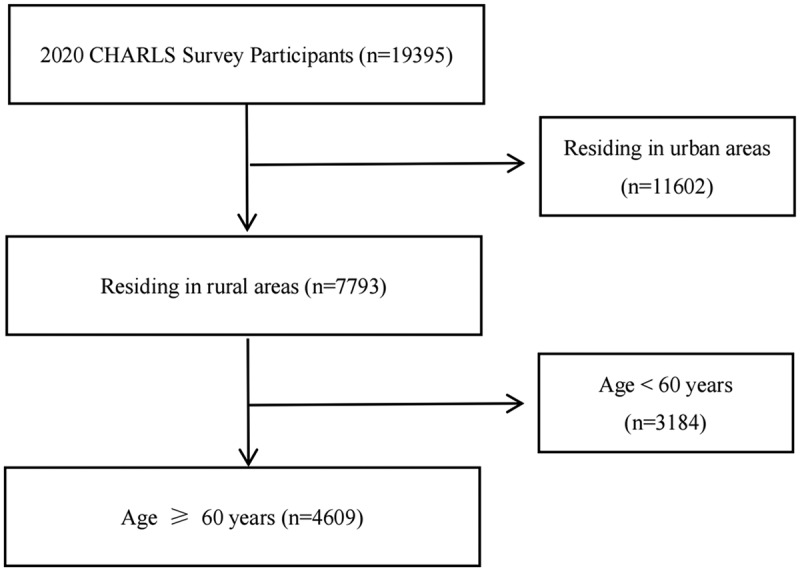
Sample screening flowchart.

Inclusion criteria were individuals aged ≥ 60 years, Chinese citizens, permanently residing in rural areas, and able to complete questionnaires independently or with assistance. Individuals with severe mental disorders, cognitive impairment, or loss of consciousness, or those involved in similar investigations, were excluded. The sample selection process is shown in Figure 2.

### Research variables

Sociodemographic covariates included gender, age, marital status, educational attainment, and household per capita consumption. Gender was coded as female or male, and marital status was coded as unmarried or married. Educational attainment was categorised as no formal education, primary school, middle school, and high school or above.

### Digital divide

Drawing on prior research on digital inequality and the measurement framework used in related studies [24,35], we operationalized the digital divide as a multidimensional construct reflecting differences in digital access, breadth of use, and practical digital skills. This approach was informed by recent studies showing that digital disadvantage among Chinese older adults can be meaningfully assessed using indicators spanning access, usage breadth, and application-related capabilities [8], and that internet skills and differentiated online activities are important dimensions of digital inequality in later life [7]. Guided by this framework and the CHARLS 2020 questionnaire, we selected three indicators: (1) digital access, proxied by past-month internet use; (2) diversity of online activities, assessed by the number of reported online activities; (3) practical digital skills, assessed by knowledge of mobile payment applications. Because CHARLS 2020 does not provide direct measures of online frequency, depth of engagement, or downstream digital benefits, this operationalisation was intended to capture multiple relevant dimensions of digital disadvantage rather than relying on a single indicator alone. After reverse coding, the three indicators were combined into a composite digital divide score, with higher values indicating greater digital disadvantage. Participants were then grouped into tertiles of this composite score (high, moderate, and low digital divide) based on the sample distribution.

### Health status

To capture overall health more comprehensively, we constructed a composite health status measure including physical functioning, mental health, and self-rated health. Physical functioning was assessed using Activities of Daily Living (ADL) across six basic functions (bathing, dressing, eating, toileting, continence, and getting in/out of bed). Participants reporting no difficulty on all six items were coded as 1, and those reporting difficulty on at least one item were coded as 0. Mental health was measured using the 10-item Center for Epidemiologic Studies Depression Scale (CESD–10), with total scores ranging from 0 to 30; scores were reverse-coded, so that higher values indicated better mental health. Self-rated health was assessed by ‘How would you rate your current health status – very good, good, fair, poor, or very poor?’ and dichotomised as very good/good (1) versus fair/poor/very poor (0), consistent with prior work [15]. After all components had been aligned in the same positive direction, each component was standardized separately, and the standardized scores were summed to generate the composite health index, with higher values indicating better overall health.

### Cognitive function

Cognitive function was assessed through face-to-face interviews and captured three domains: orientation/attention (TICS—10), episodic memory, and visuoconstruction. The TICS–10 (Telephone Interview for Cognitive Status–10) comprised serial subtraction of sevens and temporal orientation (0–10). Episodic memory was measured by immediate and delayed recall of a 10-word list (0–20). Visuoconstruction was assessed using a figure-copying task (0/1). Scores were summed to generate a total cognitive function score ranging from 0 to 31 [36].

### Social participation

Social participation in the 2020 CHARLS was measured using the item: ‘Have you participated in any of the following activities during the past month?’ [37]. Seven activities were assessed: interacting with friends; recreational activities (e.g. playing mahjong or chess); providing unpaid help to others; participating in sports or social clubs; attending community organisations; volunteering; and caring for sick or disabled individuals not living in the same household. Each endorsed activity was coded as 1 and summed to yield a total social participation score ranging from 0 to 7, with higher scores indicating greater social participation.

### Broadband connection

Broadband connection was determined by the question: ‘Is broadband Internet access available in your current place of residence?’ Responses were coded as 1 = Yes and 0 = No.

### Physical activity

Physical activity (PA) was assessed using IPAQ-based items. In 2020 CHARLS participants reported ≥ 10-min sessions of vigorous (VPA), moderate (MPA), and light (LPA) activity, including weekly frequency (1–7 days) and daily duration (≥10–<30 min, 30 min–<2 h, 2–<4 h, ≥4 h) [38]. Weekly time was calculated as frequency × daily duration. Total PA volume (PAV; MET – min/week) was computed as: PAV = 8.0×VPA +4.0×MPA +3.3 × LPA. PAV was classified as Level 1 (<600), Level 2 (600–<3000), or Level 3 (≥3000 MET – min/week) per IPAQ [39].

### Data analysis

Continuous variables are described as Mean ± SD, and categorical variables are described as n (%) Missing data were handled using multiple imputation in R (mice; 20 imputations) [40]. Further analytical details are provided in Supplementary Tables S2–S6. Hypotheses were tested using regression-based path models in SPSS 27.0 with PROCESS [41]. Before constructing the composite digital divide index, exploratory factor analysis (EFA) was used to examine the structure of candidate indicators, and Bartlett’s test of sphericity assessed factorability. Group differences were tested using t-tests or one-way ANOVA for continuous variables and chi-square tests for categorical variables. Spearman correlations were computed, with the Benjamini–Hochberg procedure controlling the false discovery rate for multiple comparisons. Regression-based path models were used to test hypothesised associations and pathways, with adjustment for gender, age, education level, marital status, and household per capita consumption. Mediation and moderation were examined using PROCESS with 5,000 bootstrap resamples and 95% confidence intervals. Given the cross-sectional design, the indirect effects were interpreted as statistical decompositions of associations rather than as evidence of temporal or causal mediation. Continuous variables in interaction terms were z-standardized. AMOS was used only to evaluate the overall fit of the observed–variable path model, whereas PROCESS was used to estimate path coefficients, indirect effects, and moderated mediation effects with bootstrap confidence intervals.

## Results

### Participant characteristics

Exploratory factor analysis indicated that the three selected indicators jointly reflected a common underlying dimension of the digital divide: all indicators loaded on a single factor, which explained 63.5% of the total variance, and Bartlett’s test of sphericity was significant (*p* < 0.001). These findings supported the use of a composite indicator to characterise the digital divide, allowing differences across multiple dimensions to be captured more comprehensively than by any single indicator alone.

Table 1 presents the characteristics of the 4,609 participants. The mean age was 70.02 ± 7.27 years, and the mean scores for cognitive function, social participation, and health status were 16.00 ± 4.88, 0.81 ± 1.03, and 22.80 ± 5.96, respectively; mean household per capita consumption was 22,188.34 ± 19,290.36. All continuous variables varied significantly across digital divide tertiles (all *p* < 0.001). Relative to the high digital divide group, the low digital divide group showed better cognitive function, greater social participation, better health status, and higher household consumption, and were younger. Gender, educational level, marital status, broadband connection, and physical activity level also differed significantly across tertiles (all *p* < 0.001).

**Table 1.** Descriptive characteristics of participants.

Variables	Total	Low Digital divide	Moderate Digital divide	High Digital divide	*P*
	*N* = 4609	(*N* = 1219)	*N* = 1382	*N* = 2008	
Continuous variables (Mean ± SD)					
Cognitive function	16.00 ± 4.88	19.45 ± 3.41	16.77 ± 4.26	13.36 ± 4.52	<0.001
Social participation	0.81 ± 1.03	1.31 ± 1.28	0.82 ± 0.95	0.48 ± 0.75	<0.001
Health status	22.80 ± 5.96	25.25 ± 4.84	23.13 ± 5.64	21.08 ± 6.23	<0.001
Age	70.02 ± 7.27	66.78 ± 5.41	70.16 ± 7.06	71.90 ± 7.70	<0.001
Household per capita consumption	22,188.34 ± 19290.36	29,437.16 ± 24243.97	22,222.47 ± 17904.00	17,764.29 ± 15019.92	<0.001
Categorical variables n (%)					
Gender					<0.001
Female	2413 (52.4)	578 (24.0)	780 (32.3)	1055 (43.7)	
Male	2196 (47.6)	641 (29.2)	602 (27.4)	953 (43.4)	
Marital status					<0.001
Unmarried	1022 (22.2)	161 (15.8)	267 (26.1)	594 (58.1)	
Married	3587 (77.8)	1058 (29.5)	1115 (31.1)	1414 (39.4)	
Educational level					<0.001
No formal education	1790 (38.8)	107 (6.0)	478 (26.7)	1205 (67.3)	
Primary school	945 (20.5)	177 (18.7)	363 (38.4)	405 (42.9)	
Middle school	991 (21.5)	413 (41.7)	312 (31.5)	266 (26.8)	
High school or above	883 (19.2)	522 (59.1)	229 (25.9)	132 (14.9)	
Broadband connection					<0.001
No	1740 (37.8)	204 (11.7)	520 (29.9)	1016 (58.4)	
Yes	2869 (62.2)	1015 (35.4)	862 (30.0)	992 (34.6)	
Physical activity					<0.001
Low	846 (18.4)	100 (11.8)	204 (24.1)	542 (64.1)	
Moderate	1635 (35.5)	503 (30.8)	480 (29.4)	652 (39.9)	
High	2128 (46.2)	616 (28.9)	698 (32.8)	814 (38.3)	

### Correlations among key variables

Table 2 shows the bivariate correlations among the key variables. Health status was positively correlated with cognitive function (ρ = 0.367) and social participation (ρ = 0.138), and negatively correlated with the digital divide (ρ = −0.293), these correlations remained significant after FDR correction (*p* < 0.001). In addition, social participation was positively correlated with cognitive function (ρ = 0.217) and negatively correlated with the digital divide (ρ = −0.328). The strongest correlation was found between cognitive function and the digital divide (ρ = −0.540).

**Table 2.** Spearman correlations among variables.

Comparison	Spearman’s ρ	*P* – value	FDR – adjusted *P* – value
Health status ~ Social participation	0.138**	<0.001	<0.001
Health status ~ Cognitive function	0.367**	<0.001	<0.001
Health status ~ Digital divide	−0.293**	<0.001	<0.001
Social participation ~ Cognitive function	0.217**	<0.001	<0.001
Social participation ~ Digital divide	−0.328**	<0.001	<0.001
Cognitive function ~ Digital divide	−0.540**	<0.001	<0.001

Note: ρ represents the Spearman correlation coefficient; *p* < 0.01. *p* – values were corrected for false discovery rate (FDR) using the Benjamini–Hochberg method.

### Mediation analysis

The observed-variable path model showed acceptable overall fit in AMOS (χ^2^ = 19.148, CFI = 0.993, TLI = 0.959, GFI = 0.998, AGFI = 0.979, RMSEA = 0.063). Statistical inference for the direct, indirect, and conditional indirect effects was based on PROCESS analyses using 5,000 bootstrap resamples.

Bootstrap analyses based on 5,000 resamples showed significant indirect associations between the digital divide and health status through both cognitive function and social participation. Specifically, the indirect effect via cognitive function was −0.349 (95% bootstrap CI: −0.410 to −0.291), accounting for 35.5% of the total effect, whereas the indirect effect via social participation was −0.076 (95% bootstrap CI: −0.117 to −0.036), accounting for 7.7% of the total effect (Table 3). Detailed estimates of the mediation effects are provided in Supplementary Table S1.

**Table 3.** Mediation effects of cognitive function and social participation.

Mediating pathway	X → M (a)	M → Y (b)	Total effect (c)	Direct effect (c′)	Indirect effect (ab)	95% bootstrap CI
Digital divide → Cognitive function → Health status	−1.145***	0.304***	−0.984***	0.636***	−0.349	[−0.410, −0.291]
Digital divide → Social participation → Health status	−0.255***	0.298***	−0.984***	−0.908***	−0.076	[−0.117, −0.036]

***p < 0.001.

### Moderation analysis

In the PROCESS Model 7 (Table 4), broadband connectivity significantly moderated the association between the digital divide and cognitive function (B = 0.092, *t* = 3.363, *p* < 0.001), with a weaker negative association among participants with broadband connectivity. In the PROCESS Model 14 (Table 4), physical activity significantly moderated the association between social participation and health status (B = −0.143, *t* = −4.498, *p* < 0.001), indicating that the association between social participation and health status was weaker among participants with higher physical activity.

**Table 4.** Moderation effects analysis.

Outcome variable	Predictor variables	R^2^	R	F	B	t
Cognitive function	Digital divide	0.374	0.612	344.038	−0.361	−14.871***
	Digital divide × Broadband connection				0.092	3.363***
Health status	Digital divide	0.095	0.308	53.509	−0.164	−6.256***
	Social participation				0.411	5.028***
	Social participation × Physical activity				−0.143	−4.498***

**Figure 3.**
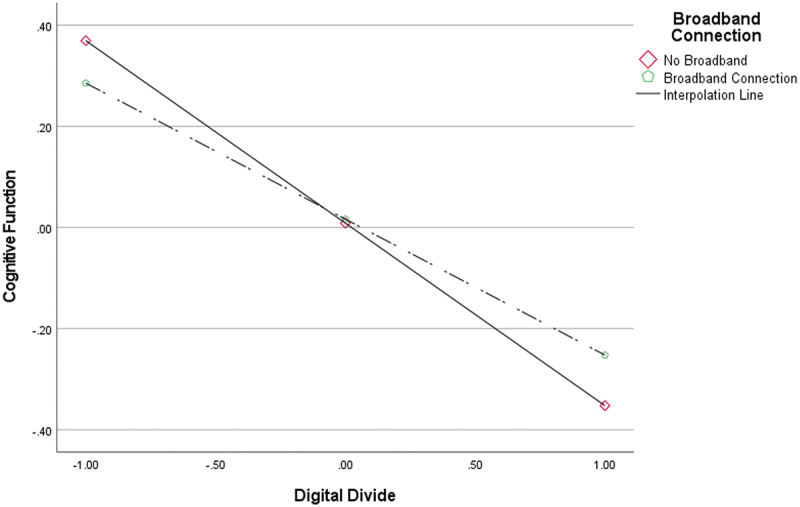
Moderating effect of broadband connection.

Figure 3 shows that the association between the digital divide and cognitive function differed by broadband connectivity. Conditional effects indicated significant negative associations in both groups, but the magnitude varied: the association was stronger among rural older adults without broadband connectivity (B = −0.361, *p* < 0.001) and weaker among those with broadband connectivity (B = −0.269, *p* < 0.001). These results suggest cross-sectional heterogeneity in the strength of the association by broadband connectivity.

**Figure 4.**
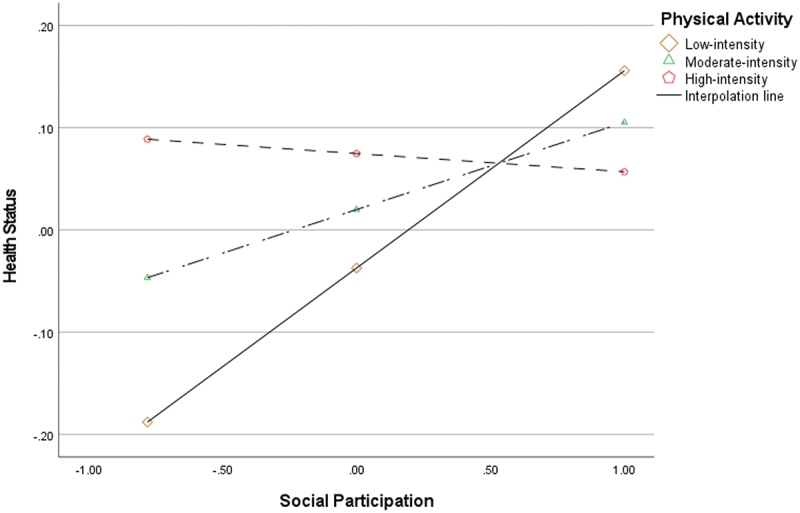
Moderating effect of physical activity.

Figure 4 shows that the association between social participation and health status differed across physical activity levels. The conditional effect of social participation was positive and significant at low physical activity (B = 0.193, *p* < 0.001) and at moderate physical activity (B = 0.085, *p* < 0.001), but was not significant at high physical activity (B = −0.018, *p* = 0.524). Together, these findings indicate that the positive association between social participation and health status was more pronounced at low-to-moderate levels of physical activity and was attenuated at higher levels.

## Discussion

Using CHARLS 2020 data, we found that a larger digital divide was associated with poorer health status among rural older adults, consistent with prior evidence linking digital inequality to health disparities [42]. Beyond the overall association, our analyses suggested statistically significant indirect associations involving cognitive function and social participation, and the strength of key associations varied by broadband connectivity and physical activity. Taken together, these findings highlight cognitive and social patterns linking digital inequality to health, as well as contextual variation by broadband access and physical activity in rural ageing populations.

Using a multidimensional operationalisation of the digital divide and a tertile grouping approach, we further found that older adults in the low digital divide group tended to report better health status, higher cognitive function, greater social participation, and higher household per capita consumption. However, the raw digital divide score distribution was highly skewed, with substantial clustering at the floor of the distribution. This suggests that the tertile grouping may partly reflect the basic distinction between internet users and non-users, rather than fully capturing more nuanced levels of multidimensional digital inequality. Therefore, these grouped comparisons should be interpreted cautiously. Conversely, participants in the high digital divide group showed lower levels across these characteristics. Furthermore, the association between social participation remained materially unchanged in a robustness check using a negative binomial model. This pattern is plausible given that increasing age is often accompanied by greater constraints in accessing and using digital technologies [43]. In rural settings, limited access to devices and digital resources may restrict opportunities to obtain information and public services, and may coincide with greater social isolation and poorer mental and physical health [44]. In addition, the observed gradient in per capita household consumption suggests that economic resources may also be closely related to digital access and use among rural older adults.

We also observed a statistically significant indirect association via cognitive function. Limited digital access and lower digital skills may reduce information exposure and participation in cognitively stimulating activities, which may constrain cognitive reserve and link digital inequality to poorer health [45]. In this context, online learning, virtual health education, and digitally enabled social interaction may be particularly relevant to maintaining cognitive function and slowing age-related decline [10,46,47]. These findings suggest that cognitively engaging forms of digital participation may be relevant to maintaining cognitive function and supporting cognitive health in later life.

At the same time, the observed cognitive pathway should not be interpreted as strictly unidirectional. An alternative explanation is that poorer health status may itself contribute to cognitive decline, and lower cognitive function may then reduce older adults’ ability, confidence, or willingness to engage with digital tools, thereby contributing to greater digital disadvantage. This interpretation is supported by longitudinal evidence showing that poorer self-rated health predicts subsequent dementia risk among middle-aged and older adults [48]. In addition, because individuals with severe cognitive problems were excluded during sample selection, the lower end of the cognitive-function distribution may have been underrepresented. Accordingly, the present findings should be interpreted as cross-sectional associations compatible with a possible cognitive pathway, rather than as evidence of a directional causal mechanism.

A second statistically significant indirect association was observed via social participation. Digital technologies may support older adults’ social lives by expanding social networks and improving the frequency and quality of interaction with family and friends [49]. Conversely, a larger digital divide may limit opportunities for social participation and reduce interaction within existing circles, which may exacerbate mental health problems and, in turn, relate to poorer overall health [10,50]. Taken together, our findings align with the view that digital inclusion may be relevant to health partly by enabling social connection and participation.

Although broadband connectivity was associated with a weaker negative association between the digital divide and cognitive function, this interaction was small in magnitude and should therefore be interpreted cautiously. One possible explanation is that, in rural settings, more stable broadband access may modestly reduce basic barriers to sustained online engagement, which could slightly weaken the negative association between the digital divide and cognitive function [26]. This contextual pattern appeared less evident when the level of the digital divide was relatively low, and rural heterogeneity in broadband availability and network quality may have further reduced observable differences. Broadband may therefore be better understood as a modest contextual condition rather than a strong moderator. Its role may be somewhat more evident when digital inequality is more pronounced, but the practical significance of this finding appears limited, and further replication is needed before drawing stronger conclusions.

At the same time, the moderating pattern involving physical activity should be interpreted cautiously. Although the positive association between social participation and health status appeared weaker at higher levels of physical activity, this finding is not straightforward and may reflect several context-specific factors rather than a simple behavioural mechanism. In rural older populations, higher physical activity may partly reflect labour-intensive daily work rather than leisure-time exercise, and its implications for health may therefore differ from those of structured or recreational activity. Prior evidence suggests more consistent benefits of low-to-moderate-intensity activity for outcomes such as self-rated health, well-being, and related physical and mental health indicators [29,47,51]. Against this background, the present finding may suggest that the health-related role of social participation varies across different physical activity contexts.

## Strength and limitations

This study used a large, nationally representative sample, enhancing the generalisability of the findings. It also examined both cognitive and social pathways as well as contextual modification by broadband connectivity and physical activity.

This study has several limitations. First, due to the cross-sectional design, temporal ordering among the digital divide, cognitive function, social participation, and health status could not be established; thus, the indirect effects should be interpreted with caution, and reverse causation cannot be ruled out. Future studies using longitudinal or multi-wave CHARLS data are needed to clarify directionality. Second, digital use was assessed through proxy measures rather than direct behavioural indicators. Third, the results may have been influenced by self-report bias. Finally, although we adjusted for key sociodemographic characteristics and examined core pathways via cognitive function and social participation, unmeasured mechanisms and residual confounding may still exist, such as chronic disease burden, access to health services, living arrangements, and community-level resource differences.

## Implications for policy and practice

Addressing digital exclusion is important for reducing health inequities among rural older adults. In this study, the digital divide reflected multidimensional disadvantages across access, breadth of use, and practical digital skills, suggesting that interventions should move beyond simple connectivity and also address the ways in which older adults actually use and navigate digital tools. At the structural level, expanding reliable and affordable broadband coverage in rural communities may create conditions for sustained online engagement and reduce barriers to using digital services. At the individual level, accessible, skills-focused support that strengthens digital health literacy, such as learning how to search for, appraise, and use online health information and services, may be particularly beneficial for those with the greatest digital barriers.

Our findings also highlight the importance of behavioural context. Because the moderating pattern involving physical activity should be interpreted cautiously, the implication is not that higher activity is harmful, but rather that the context, feasibility, and sustainability of activity may matter when social participation translates into health-related benefits among rural older adults. Community-based programmes that combine social connection with feasible and sustainable activities, such as walking groups and everyday movement, may therefore warrant further attention.

In community and primary-care settings, health workers, including nurses, are well positioned to identify digital barriers, provide tailored guidance on using digital health resources, and support routine self-management. Together, infrastructure improvement, digital health literacy support, and feasible physical activity promotion represent complementary, equity-oriented priorities for supporting healthy ageing in rural populations.

## Conclusion

This cross-sectional study found that a larger digital divide was associated with poorer health status among rural older adults. Cognitive function and social participation may help explain this association, suggesting possible cognitive and social pathways. We also observed contextual variation, with the strength of these associations varying by broadband connectivity and physical activity. These findings provide cross-sectional evidence to inform future research, rather than confirmation of causal pathways. Longitudinal and intervention studies are needed to clarify temporal ordering and further examine these relationships.

## Supplementary Material

Supplementary Materials.docx

## Data Availability

The data analyzed in this study are publicly available from the China Health and Retirement Longitudinal Study (CHARLS) project website (https://charls.charlsdata.com.).

## References

[1] Du P, Yang H. China’s population ageing and active ageing. In: Morrow-Howell N, Mui AC, editors. Productive engagement in later life. New York: Routledge; 2014. p. 33–12.

[2] Li L, Jin G, Guo Y, et al. Internet access, support, usage divides, and depressive symptoms among older adults in China: a nationally representative cross-sectional study. J Affect Disord. 2023;323:514–523. doi: 10.1016/j.jad.2022.12.001 36496102

[3] Hargittai E, Dobransky K. Old dogs, new clicks: digital inequality in skills and uses among older adults. Can J Commun. 2017;42:195–212. doi: 10.22230/cjc.2017v42n2a3176

[4] Besser H. The next digital divides. Teach Change LA. 2001;1(2).

[5] Friemel TN. The digital divide has grown old: determinants of a digital divide among seniors. New Media Soc. 2016;18(2):313–331. doi: 10.1177/1461444814538648

[6] Schehl B, Leukel J, Sugumaran V. Understanding differentiated internet use in older adults: a study of informational, social, and instrumental online activities. Comput Hum Behav. 2019;97:222–230. doi: 10.1016/j.chb.2019.03.031

[7] Mu A, Liu Z. Assessing the impact of internet skills on depressive symptoms among Chinese middle-aged and older adults: cross-sectional instrumental variables analysis. JMIR Aging. 2024;7:e50880. doi: 10.2196/50880 38533782 PMC11004627

[8] Feng Y, Pu K, Liu C. Digital divide and healthcare service utilization among older adults with chronic liver disease in China: a nationwide cross-sectional study. Sci Rep. 2026;16:4448. doi: 10.1038/s41598-025-34523-0 41559268 PMC12864921

[9] Choi NG, DiNitto DM. The digital divide among low-income homebound older adults: internet use patterns, eHealth literacy, and attitudes toward computer/internet use. J Med Internet Res. 2013;15(5):e93. doi: 10.2196/jmir.2645 23639979 PMC3650931

[10] Liu J, Su W. Impacts of the silver digital divide on physical and mental health of the elderly population: based on data from three-year China Family Panel Studies (CFPS). Popul J. 2022;44(6):53–68.

[11] Liu J, Wang L, Tan J. Dementia in China: current status. Neurology. 2013;81(12):1077–1078. doi: 10.1212/WNL.0b013e3182a4a3cb 24042573

[12] Saenz JL, Downer B, Garcia MA, et al. Cognition and context: rural–urban differences in cognitive aging among older Mexican adults. J Aging Health. 2018;30(6):965–986. doi: 10.1177/0898264317703560 28553815 PMC5623618

[13] Wang Y, Wu Z, Duan L, et al. Digital exclusion and cognitive impairment in older people: findings from five longitudinal studies. BMC Geriatr. 2024;24(1):406. doi: 10.1186/s12877-024-05026-w 38714939 PMC11077883

[14] Yu D, Fiebig DG. Internet use and cognition among middle-aged and older adults in China: a cross-lagged panel analysis. J Econ Ageing. 2020;17:100262. doi: 10.1016/j.jeoa.2020.100262

[15] Liu J, Rozelle S, Xu Q, et al. Social engagement and elderly health in China: evidence from the China Health and Retirement Longitudinal Survey (CHARLS). Int J Environ Res Public Health. 2019;16(2):278. doi: 10.3390/ijerph16020278 30669415 PMC6352065

[16] Cotten SR, Anderson WA, McCullough BM. Impact of internet use on loneliness and contact with others among older adults: cross-sectional analysis. J Med Internet Res. 2013;15(2):e2306. doi: 10.2196/jmir.2306 PMC3636305 23448864

[17] Warburton J, Cowan S, Bathgate T. Building social capital among rural, older Australians through information and communication technologies: a review article. Australas J Ageing. 2013;32(1):8–14. doi: 10.1111/j.1741-6612.2012.00634.x 23521728

[18] Stern Y. Cognitive reserve in ageing and Alzheimer’s disease. Lancet Neurol. 2012;11(11):1006–1012. doi: 10.1016/S1474-4422(12)70191-6 23079557 PMC3507991

[19] Rains SA. Health at high speed: broadband internet access, health communication, and the digital divide. Commun Res. 2008;35(3):283–297. doi: 10.1177/0093650208315958

[20] Cheng F, Cao L. Broadband access, internet use, and general cognition in middle-aged and older adults: longitudinal study. JMIR Aging. 2026;9(1):e75947. doi: 10.2196/75947 41875201 PMC13012894

[21] Kyriazis M, Kiourti E. Video games and other online activities may improve health in ageing. Front Med. 2018;5:8. doi: 10.3389/fmed.2018.00008 PMC5796895 29435449

[22] Duffner LA, DeJong NR, Jansen JF, et al. Associations between social health factors, cognitive activity and neurostructural markers for brain health: a systematic literature review and meta-analysis. Ageing Res Rev. 2023;89:101986. doi: 10.1016/j.arr.2023.101986 37356551

[23] Yu X, Mu A, Wu X, et al. Impact of internet use on cognitive decline in middle-aged and older adults in China: longitudinal observational study. J Med Internet Res. 2022;24(1):e25760. doi: 10.2196/25760 35072642 PMC8822429

[24] Van Dijk JA. Digital divide research, achievements and shortcomings. Poetics. 2006;34(4–5):221–235. doi: 10.1016/j.poetic.2006.05.004

[25] Yang Q, Sun Z, Wang D, et al. Frontiers and comparisons of digital village research in China and internationally from a geographical perspective. Prog Geogr. 2025;44(4):657–669. doi: 10.18306/dlkxjz.2025.04.001

[26] Grzybowski L, Liang J. Estimating demand for fixed-mobile bundles and switching costs between tariffs. Inf Econ Policy. 2015;33:1–10. doi: 10.1016/j.infoecopol.2015.08.002

[27] Bull FC, Al-Ansari SS, Biddle S, et al. World Health Organization 2020 guidelines on physical activity and sedentary behaviour. Br J Sports Med. 2020;54(24):1451–1462. doi: 10.1136/bjsports-2020-102955 33239350 PMC7719906

[28] Blair CK, Morey MC, Desmond RA, et al. Light-intensity activity attenuates functional decline in older cancer survivors. Med Sci Sports Exerc. 2014;46(7):1375–1383. doi: 10.1249/MSS.0000000000000241 24389524 PMC4061152

[29] Buman MP, Hekler EB, Haskell WL, et al. Objective light-intensity physical activity associations with rated health in older adults. Am J Epidemiol. 2010;172(10):1155–1165. doi: 10.1093/aje/kwq249 20843864 PMC3004766

[30] Corbett DB, Rejeski WJ, Tudor-Locke C, et al. Social participation modifies the effect of a structured physical activity program on major mobility disability among older adults: results from the LIFE study. The Journals Gerontology. 2018;73(8):1501–1513. doi: 10.1093/geronb/gbx051 PMC6178963 28482106

[31] Stevens M, Cruwys T. Membership in sport or exercise groups predicts sustained physical activity and longevity in older adults compared to physically active matched controls. Ann Behav Med. 2020;54(8):557–566. doi: 10.1093/abm/kaaa003 32044986

[32] Fain RS, Hayat SA, Luben R, et al. Effects of social participation and physical activity on all-cause mortality among older adults in Norfolk, England: an investigation of the EPIC-Norfolk study. Public Health. 2022;202:58–64. doi: 10.1016/j.puhe.2021.10.017 34894534

[33] Cui Y, He Y, Xu X, et al. Cultural capital, the digital divide, and the health of older adults: a moderated mediation effect test. BMC Public Health. 2024;24(1):302. doi: 10.1186/s12889-024-17831-4 38273305 PMC10811880

[34] Yi J, Yoon JY, Won CW, et al. The roles of health literacy and social support in the association between smartphone ownership and frailty in older adults: a moderated mediation model. BMC Public Health. 2024;24(1):1064. doi: 10.1186/s12889-024-18163-z 38632509 PMC11037091

[35] Hsieh JP, Rai A, Keil M. Understanding digital inequality: comparing continued use behavioral models of the socio-economically advantaged and disadvantaged. Mis Q. 2008;32(1):97–126. doi: 10.2307/25148830

[36] Cao L, Zhao Z, Ji C, et al. Association between solid fuel use and cognitive impairment: a cross-sectional and follow-up study in a middle-aged and older Chinese population. Environ Int. 2021;146:106251. doi: 10.1016/j.envint.2020.106251 33248346

[37] Ge H, Dong S, Su W, et al. Relationship between social participation and depressive symptoms in patients with multimorbidity: the chained mediating role of cognitive function and activities of daily living. BMC Public Health. 2024;24(1):1844. doi: 10.1186/s12889-024-19157-7 38987791 PMC11234698

[38] Ding M, Jia N, Zhou Y, et al. The dose–response relationships of different dimensions of physical activity with daily physical function and cognitive function in Chinese adults with hypertension: a cross-sectional study. Int J Environ Res Public Health. 2021;18(23):12698. doi: 10.3390/ijerph182312698 34886423 PMC8657437

[39] Li X, Zhang W, Zhang W, et al. Level of physical activity among middle-aged and older Chinese people: evidence from the China Health and Retirement Longitudinal Study. BMC Public Health. 2020;20(1):1682. doi: 10.1186/s12889-020-09671-9 33172439 PMC7653852

[40] Van Buuren S. Multiple imputation of discrete and continuous data by fully conditional specification. Stat Methods Med Res. 2007;16(3):219–242. doi: 10.1177/0962280206074463 17621469

[41] Hayes AF. Partial, conditional, and moderated moderated mediation: quantification, inference, and interpretation. Commun Monog. 2018;85(1):4–40. doi: 10.1080/03637751.2017.1352100

[42] Yang R, Gao S, Jiang Y. Digital divide as a determinant of health in the US older adults: prevalence, trends, and risk factors. BMC Geriatr. 2024;24:1027. doi: 10.1186/s12877-024-05612-y 39709341 PMC11662839

[43] Lee B, Chen Y, Hewitt L. Age differences in constraints encountered by seniors in their use of computers and the internet. Comput Hum Behav. 2011;27(3):1231–1237. doi: 10.1016/j.chb.2011.01.003

[44] Sen K, Prybutok G, Prybutok V. The use of digital technology for social wellbeing reduces social isolation in older adults: a systematic review. SSM Popul Health. 2022;17:101020. doi: 10.1016/j.ssmph.2021.101020 35024424 PMC8733322

[45] Li Y, Liu C, Sun J, et al. The digital divide and cognitive disparities among older adults: community-based cohort study in China. J Med Internet Res. 2024;26:e59684. doi: 10.2196/59684 39602795 PMC11635332

[46] Almeida OP, Yeap BB, Alfonso H, et al. Older men who use computers have lower risk of dementia. PLoS One. 2012;7(8):e44239. doi: 10.1371/journal.pone.0044239 22937167 PMC3429429

[47] Healy GN, Dunstan DW, Salmon J, et al. Objectively measured light-intensity physical activity is independently associated with 2-h plasma glucose. Diabetes Care. 2007;30(6):1384–1389. doi: 10.2337/dc07-0114 17473059

[48] Stephan Y, Sutin AR, Luchetti M, et al. Self-rated health and incident dementia over two decades: replication across two cohorts. J Psychiatr Res. 2021;143:462–466. doi: 10.1016/j.jpsychires.2021.06.036 34311955

[49] Wright K. Computer-mediated social support, older adults, and coping. J Commun. 2000;50(3):100–118. doi: 10.1111/j.1460-2466.2000.tb02855.x

[50] Vroman KG, Arthanat S, Lysack C. “Who over 65 is online?” Older adults’ dispositions toward information communication technology. Comput Hum Behav. 2015;43:156–166. doi: 10.1016/j.chb.2014.10.018

[51] Loprinzi PD. Objectively measured light and moderate-to-vigorous physical activity is associated with lower depression levels among older US adults. Aging Ment Health. 2013;17(7):801–805. doi: 10.1080/13607863.2013.801066 23731057

