# Supplementary material for: Digital divide and health status among rural older adults in China: evidence from CHARLS
Source: Glob Health Action. 2026 Jun 2;19(1):2679945. doi: 10.1080/16549716.2026.2679945 (PMC13231809; doi:10.1080/16549716.2026.2679945)
Supplement: Supplementary Materials.docx [file ZGHA_A_2679945_SM9959.docx]

**Supplemental Materials**

[Table S1. Mediation effects of cognitive function and social participation on the association between digital divide and health status 2](#_Toc5299)

[Table S2. Missing-data proportions and multiple imputation details for analysis variables 3](#_Toc18209)

[Table S3. Sensitivity analysis for the mediating effect of cognitive function 4](#_Toc31321)

[Table S4. Sensitivity analysis for the mediating effect of social participation 5](#_Toc27097)

[Table S5. Sensitivity analysis for the moderating effect of broadband connectivity on the pathway from digital divide to cognitive function 6](#_Toc1488)

[Table S6. Sensitivity analysis for the moderating effect of physical activity on the pathway from social participation to health status 7](#_Toc8378)

**Table S1. Mediation effects of cognitive function and social participation on the association between digital divide and health status**

| **Mediator** | **N** | **X→M (a)** | **M→Y** | **Total effect** | **Direct effect** | **Indirect effect** | **95% CI** |
| --- | --- | --- | --- | --- | --- | --- | --- |
| Cognitive function | 4609 | -1.145*** | 0.304*** | -0.984*** | -0.636*** | -0.349 | -0.410 to -0.291 |
| Social participation | 4609 | -0.255*** | 0.298*** | -0.984*** | -0.908*** | -0.076 | -0.117 to -0.036 |

Note. Both pathways were tested in separate single-mediator models rather than in one parallel mediation model（***p < 0.001）.

**Table S2. Missing-data proportions and multiple imputation details for analysis variables**

| **Variable** | **Missing (%)** | **Imputation method** | **Remarks** |
| --- | --- | --- | --- |
| Sex | 0 | Not applicable | No missing data |
| Marry | 0 | Not applicable | No missing data |
| Age | 0 | Not applicable | No missing data |
| Education | 0 | Not applicable | No missing data |
| Digital divide | 0.52% | Predictive mean matching | Imputed |
| Broadband connectivity | 0.69% | Binary logistic regression | Imputed |
| Social participation | 0.50% | Predictive mean matching | Imputed |
| Health status | 19.89% | Predictive mean matching | Imputed |
| Cognitive function | 30.77% | Predictive mean matching | Imputed |
| Per capita consumption | 30.33% | Predictive mean matching | Imputed |

Note. Missing data were handled by multiple imputation in R (mice; 20 imputations) under a missing-at-random assumption, with variable-type-specific methods applied to continuous and binary variables.

**Table S3. Sensitivity analysis for the mediating effect of cognitive function**

| **Mediating pathway** | **X→M (a)** | **M→Y (b)** | **Direct effect (c′)** | **Indirect effect (ab)** | **95% bootstrap CI** |
| --- | --- | --- | --- | --- | --- |
| Digital divide → Cognitive function → Health status | 1.555*** | 0.252*** | 1.137*** | 0.392 | [0.269, 0.531] |

Note. Results were based on complete-case data without imputation. Indirect effects were estimated using 5,000 bootstrap resamples.
**p < 0.001.

**Table S4. Sensitivity analysis for the mediating effect of social participation**

| **Mediating pathway** | **X→M (a)** | **M→Y (b)** | **Direct effect (c′)** | **Indirect effect (ab)** | **95% bootstrap CI** |
| --- | --- | --- | --- | --- | --- |
| Digital divide → Social participation → Health status | 0.514*** | 0.293** | 1.576*** | 0.150 | [0.047, 0.257] |

Note. Results were based on complete-case data without imputation. Indirect effects were estimated using 5,000 bootstrap resamples.
**p < 0.001.

**Table S5. Sensitivity analysis for the moderating effect of broadband connectivity on the pathway from digital divide to cognitive function**

| **Moderation pathway** | **Interaction term** | **b** | **SE** | **t** | **p** | **95% CI** |
| --- | --- | --- | --- | --- | --- | --- |
| Digital divide × Broadband connection→ Cognitive function | Digital divide × Broadband connection | 0.096 | 0.042 | 2.276 | 0.023 | [0.013, 0.179] |

Note. Results were based on complete-case data without imputation.

**Table S6. Sensitivity analysis for the moderating effect of physical activity on the pathway from social participation to health status**

| **Moderation pathway** | **Interaction term** | **b** | **SE** | **t** | **p** | **95% CI** |
| --- | --- | --- | --- | --- | --- | --- |
| Social participation × Physical activity → Health status | Social participation × Physical activity | -0.071 | 0.027 | -2.607 | 0.009 | [-0.125, -0.018] |

Note. Results were based on complete-case data without imputation.
